# Optical Scattering Measurements of Laser Induced Damage in the Intraocular Lens

**DOI:** 10.1371/journal.pone.0031764

**Published:** 2012-02-10

**Authors:** Bastiaan Kruijt, Thomas J. T. P. van den Berg

**Affiliations:** Netherlands Institute for Neuroscience, Royal Netherlands Academy for Arts and Sciences, Amsterdam, The Netherlands; Washington University School of Medicine, United States of America

## Abstract

This study optically determines whether the amount of light scatter due to laser-induced damage to the intraocular lens (IOL) is significant in relation to normal straylight values in the human eye. Two IOLs with laser-induced damage were extracted from two donor eyes. Each IOL had 15 pits and/or cracks. The surface area of each pit was measured using a microscope. For 6 pits per intraocular lens the point spread function (PSF) in terms of straylight was measured and the total straylight for all 15 pits was estimated. The damage in the IOLs was scored as mild/moderate. The total damaged surface areas, for a 3.5 mm pupil, in the two IOLs were 0.13% (0.0127 mm^2^) and 0.66% (0.064 mm^2^), respectively. The angular dependence of the straylight caused by the damage was similar to that of the normal PSF. The total average contribution to straylight was log(s) = −0.82 and −0.42, much less than the straylight value of the normal eye.

The straylight due to normal levels of laser induced damage of the IOL is much lower than normal straylight values found clinically for the normal eye and may therefore be considered not significant.

## Introduction

Posterior capsule opacification (PCO) is the most common complication of cataract surgery with an incidence of 20–40% within 2–5 years after surgery [1]. Treatment of PCO is done by Nd:YAG laser capsulotomy, which creates an opening in the posterior capsule.

One of the complications following Nd:YAG capsulotomy for PCO is laser induced damage to the intraocular lens (IOL). This shows up in the form of optic pits and/or cracks in the IOL material. Factors contributing to possible laser-induced damage include the IOL damage threshold, i.e. the energy level needed to induce damage with a 50% incidence [2], which is dependent on the IOL material. For example, the damage threshold for silicone IOLs is lower than for polymethylacralate (PMMA) [2], [3]. Whether the laser damage threshold for the IOL is reached depends on the laser power output setting and the setting of the laser focal point [4].

The incidence of laser-induced damage following Nd:YAG capsulotomy is 4–40% [4], [5], and ranges from mild to severe damage. *In vitro* studies show that laser-induced damage causes no significant decrease in optical resolution [6], [7], which explains the observation that visual acuity does not tend to be affected *in vivo* [6], [8]. However, these studies did suggest that laser damage may cause glare [5], [6], [9], which was also shown in other studies [10], [11]. Although severe damage is rare, the IOL is explanted [10] in cases where IOL damage seriously affects visual function. Since (disability) glare is quantified by means of straylight according to the CIE definition [12], [13], this would show up as an increase in straylight due to the laser-induced damage. Straylight corresponds to the outer part of the point spread function (PSF) from roughly 1°–90° [14] and can be assessed clinically with the C-Quant instrument. Straylight is expressed as the ‘straylight parameter’ *s* or the logarithm of *s* (log(*s*)) and is, in addition to visual acuity, an important aspect of visual function.

In the course of an in vitro study on pseudophakic donor eyes [15] we came across a few specimens with laser-induced damage to the IOL. We used this opportunity to optically study light scattering from these damages. The results were expressed in straylight values so they could be comparable to *in vivo* straylight values. The aim of this study is to quantitatively determine whether the potential increase in straylight due to laser-induced damage to the IOL is significant in relation to normal straylight values.

## Materials and Methods

Pseudophakic donor eyes were obtained from the Cornea Bank Amsterdam (CBA), with the corneoscleral disc already removed for transplantation purposes. In two of these IOLs laser-induced damage was observed, but this damage was considered fairly typical, serious, but not severe.

Two slightly different surgical techniques were used to remove the IOL and capsular bag [15]. The iris was gently removed, after which (1) for IOL-I an incision parallel to the limbus was made (leaving the zonular fibers and ciliary body intact and attached to the capsular bag), and (2) for IOL-II the zonula fibers were cut. Using a spoon-shaped spatula, the IOL and capsule, hereafter referred to as ‘the sample’, were carefully lifted from the bulbus while removing any vitreous attachment at the same time. The sample was rinsed in PBS to remove any free iris pigment and vitreous attachment.

**Figure 1:**
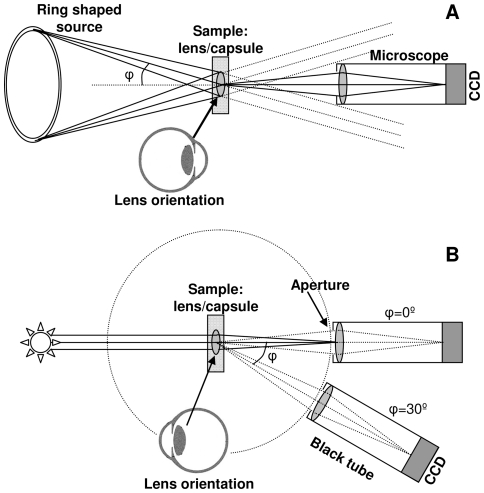
Schematic representation of a) the macroscope setup with a dark field ring illuminator; and b) the setup to quantitatively measure scattered light under different angles.

The sample was placed on a little ring in a Petri dish and submerged in PBS for qualitative examination using a dark field microscope setup (Stemi SV11, Zeiss, Jena, Germany) with dark field ring illuminator (KL 1500 LCD, Zeiss, Jena, Germany). Images were acquired using a camera (DSC-S75, Sony, The Netherlands) mounted onto the macroscope (figure 1a). Dark field images only show light that is scattered from structures in the sample. In other words when there is no scattering the field has no light intensity and appears black, in contrast to scattering structures that show an intensity value dependent on the strength of scattering. The laser-induced damage was scored on a 3 point scale (mild, moderate, or severe) according to Mamalis et al. [4], using a microscope. Where mild damage is superficial damage; moderate damage is more extensive than mild damage and includes craters and small cracks; severe damage includes severe cracking and crevice formation, and the damage extents to having influence on the shape of the lens optic [4]. In addition, the surface area of each pit was determined using a microscope (Axioskop, Zeiss, Jena, Germany) with 100× original magnification.

The amount of light scattered from the sample under different angles was quantitatively measured with a setup as described previously (figure 1b) [15], [16]. In short, the sample is placed in a clean cuvet (s<10^−1^) with phosphate-buffered saline (PBS). The sample is uniformly illuminated with green light using a narrow band interference filter, 561 nm center wavelength with a full width half maximum of 10 nm, in combination with a halogen light source. Quantitative measurements of scattered light from the illuminated sample are acquired with a CCD camera (NTE/CCD 512-TKB, Princeton Instruments Inc, Princeton NJ, USA) at different angles, corrected for the effects of refraction and reflection at the liquid-air interface, from 3° to 22°. Based on the measured values at different angles the PSF can be determined [17]. The results are presented as valid for the PSF in the in vivo situation, using the straylight parameter defined as *s* = *θ* ^2^ *PSF*, in which *θ* is the visual angle in degrees.

Light scattering by a pit or crack in the IOL will appear as an increase in the outer part of the PSF in addition to other sources of light scattering. To obtain pure values for laser damage, small local regions of interest (ROI), including the damaged area, were analyzed, sticking as close as possible to the observable pit and expressed as straylight parameter (for the ROI). For both IOLs 6 out of 15 pits were selected that were completely free from surrounding artifacts such as PCO remnants or deposits on the IOL. These 6 pits were representative for all pits based on the damage scoring and the amount of surface area affected per pit. The surface area of the ROIs ranged from 0.003 to 0.026 mm^2^, and was determined using a threshold function (the threshold value was chosen arbitrarily) in a custom made Matlab program. To determine the effect for visual function it is necessary to investigate the PSF as it would be for the complete pupil. Hence, for the 6 pits per IOL the individual contribution to overall straylight was determined for an area within a diameter of 3.5 mm by multiplying the straylight parameter with the ratio between ROI surface area and pupil surface area. To arrive at a result for all pits in the IOL the linear average of the 6 pits measured was multiplied by the total number of pits (15), a valid method if the 6 pits measured are representative of all.

## Results

**Figure 2:**
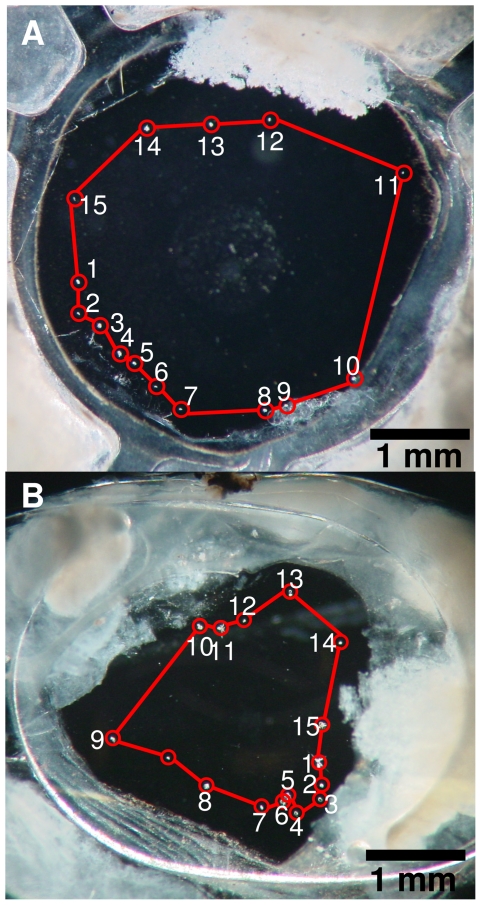
Dark field images of the IOLs acquired with the macroscope setup. Dark regions represent low scattering and light areas represent high scattering. In both images the laser pits and cracks are demarcated. a) IOL with 15 damaged areas with a circumference of approximately 3.5 mm; and b) IOL also with 15 damaged areas, but a circumference of 2–2.5 mm.

Figures 2a and b show the two IOLs with damage or pits (encircled) after ND:YAG capsulotomy. Figure 2a shows IOL-I with 15 pits all scored as mild with a total surface area of 0.0127 mm^2^. The pattern is roughly circular with a diameter of 3.5 mm. The total damaged surface area of the IOL is 0.13% of the total lens surface area within a diameter of 3.5 mm ( = 9.6 mm^2^). Figure 2b shows IOL-II also with 15 pits with a total surface area of 0.064 mm^2^, of which several were scored as moderate. In IOL-II some pits show damage patterns that extend in depth completely through the IOL. Here the circular pattern is smaller than observed in the IOL-I, 2–2.5 mm. Within a diameter of 3.5 mm, the total damaged surface area to the IOL is 0.66% of the total lens surface area.

**Figure 3:**
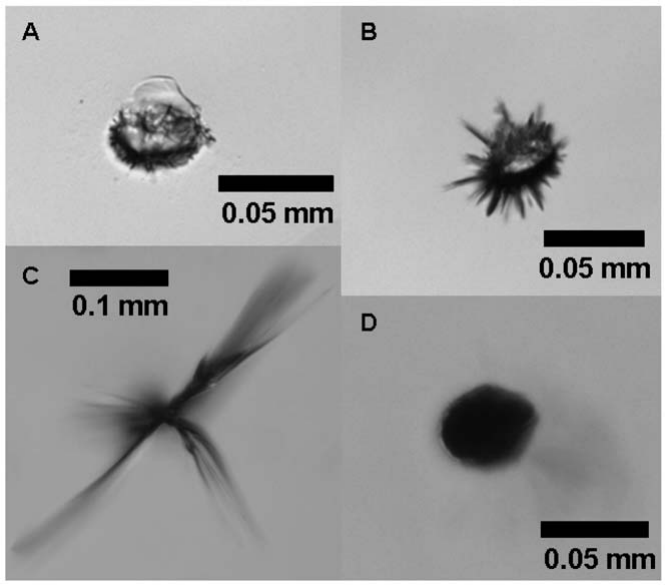
Microscope magnifications of several laser induced damaged areas in the IOLs from **figure 2**. a) area 3 in figure 2a; b) area 5 in figure 2a; c) area 1 in figure 2b; d) area 4 in figure 2b. Laser-induced damage was scored as mild damage except for c) which was scored as moderate damage.

Figure 3 shows detailed images on individual pits as depicted in figures 1 a and b. 100× original magnification shows that there are more or less round pits as well as pits with cracks of various sizes. This type of laser-induced damage has been described previously [4], [9], [18]. The damage of the pit in figure 3c was deemed moderate, the other examples were scored as mild. For determination of the surface area, shady areas around the pits or cracks were included since these indicate damage deeper in the IOL, which would also have an effect for the *in vivo* situation.

**Figure 4:**
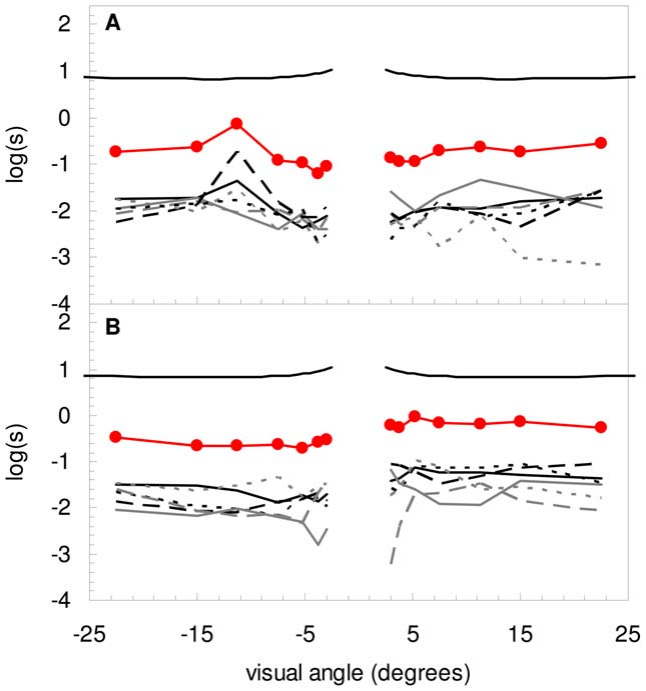
PSF of 6 individual pits (bottom 6 curves in each figure) for the IOL of a) **figure 2a and b**) **figure 2b**. The line with closed circle symbols represents the estimated total contribution of all pits in the IOL. The top PSF illustrates the PSF-model, according to the CIE, of a 35–year-old individual.

Figures 4a and b show the individual contributions to the PSF (in terms of straylight) for the 6 measured pits for both IOLs (lower 6 curves) considering a pupil diameter of 3.5 mm. The total contribution to straylight of all pits (line with closed circle symbols) is estimated by calculating the average PSF of the 6 measured pits and multiplying this by the total number of pits, in both cases 15. The average contribution of straylight over the whole angular range are log(s) = −0.82 (range −1.24 to −0.14) and −0.41 (range −0.71 to −0.04), respectively. To illustrate this, the PSF-model of a 35 year old individual, according to the CIE [19], is shown with an average straylight value over this angular range of log(s) = +0.93. Note that the angular dependence of the 6 pits is similar to the PSF-model.

## Discussion

The aim of this study was to quantify the effect of laser damage to the IOL following Nd:YAG capsulotomy on the PSF and to determine the significance of this effect. Primary findings are (1) that the angular dependence of laser damage is similar to the normal PSF, and (2) that measured values of laser damage are small compared to the normal PSF.

The damage as found in the two IOLs was considered representative for the commonly observed laser damage in IOLs, which was scored mild to moderate. These are the most common types of damage observed; the incidence of severe damage is rare [4].

According to the literature, there is no significant decrease in visual acuity due to laser damage [6]–[8], except when the IOL is severely damaged [10]. This is understandable when we consider the effect of scattering on the spatial contrast sensitivity function (CSF) (figure 9 in [13]). The highest detectable spatial frequency corresponds with visual acuity. Straylight exerts its effect over the whole range of spatial frequencies, which results in a reduction in contrast. However, the decrease in contrast is much smaller than the increase in straylight [13]. Combined with the steep decrease of the CSF at high spatial frequencies, little influence on the highest detectable spatial frequency, i.e. visual acuity, is to be expected [13]. For example, when in an extreme case 10% of the IOL surface area suffers from laser induced damage, which is a vast extrapolation and unrealistic in practice, and we only consider the effects of damage on straylight, the CSF lowers by 10% at most. The corresponding visual acuity would be lower by a fraction of that figure. Due to the steep decrease of the CSF at high frequencies, with a slope of about 10, the highest detectable spatial frequency would decrease by 1%. In terms of visual acuity expressed in decimals, this would mean a decrease from VA = 1 to VA = 0.99, which is clinically not significant (nor measurable). Only if the damage is such as to cause deformation to the overall shape of the IOL can visual acuity effects be expected.

The optimal size for posterior capsulotomy is stated to be at least equal to the size of the scotopic pupil of the patient but to remain within the border of the IOL [20]. In such a case possible laser-induced damage to the IOL would be located outside the pupil area and would cause no hindrance whatsoever. However, laser-induced damage could fall within the size of the pupil and may cause an increase in straylight, which would depend on the individual straylight intensity of the damage and the ratio between total damaged surface and pupil area. The IOLs presented in this study show circular YAG patterns, but other patterns are also used, such as a cruciate pattern [21]. When damage occurs the pattern itself has little influence, what does matter is the amount of damage and the location, whether it is in or outside the pupil opening.

For the two IOLs in this study an area within a diameter of 3.5 mm was used. Results show that the angular dependence of the straylight caused by the damage is similar to that of the normal PSF. Hence, like for the normal PSF, it is possible to describe the PSF of laser damage in a single average value. Average values for total straylight contribution over the whole angular range were shown to be −0.82 and −0.41, respectively. These values are a factor of 50 and 20 lower, respectively, than the total normal straylight value of the healthy eye of a young individual (log(s)≈0.9) [22]. Hence, laser-induced damage to the IOL of log(s) = −0.41 would increase the straylight from, for example, normal log(s) = 0.9 to log(s) = 0.92. Individual straylight sources can be linearly accumulated to obtain a total straylight value [23]. The latter is the result of the linear sum of the two straylight values ( = 10^0.9^+10^−0.41^). This increase is very small compared to clinical straylight measurements of the normal eye using the C-Quant straylight meter, and may be considered not significant. In conclusion, normal levels of laser-induced damage of the IOL cause no significant increase in straylight.
